# Validating the Italian Version of the Disgust and Propensity Scale-Revised

**DOI:** 10.3389/fpsyg.2017.00765

**Published:** 2017-05-12

**Authors:** Riccardo M. Martoni, Paola M. V. Rancoita, Clelia Di Serio, Chiara Brombin

**Affiliations:** ^1^Department of Clinical Neurosciences, IRCCS San Raffaele TurroMilan, Italy; ^2^University Center for Statistics in the Biomedical Sciences, Vita-Salute San Raffaele UniversityMilan, Italy

**Keywords:** disgust aspects, disgust propensity and sensitivity, DPSS-R, factor analysis for ordinal data, validation study

## Abstract

The aim of this work is to evaluate the factor structure and psychometric properties of the Italian version of the Disgust Propensity and Sensitivity Scale-Revised (DPSS-R, 16 items) in two samples taken from the general population. In the first study, 285 participants completed the DPSS-R questionnaire through a web-based survey. Exploratory factor analysis for ordinal Likert-type data supported the existence of four underlying factors, reflecting *self-focused disgust*, *disgust propensity*, *somatic anxiety* and *disgust sensitivity*. In the second study, an independent sample of 293 participants was enrolled as a test set to validate the factor structure obtained in the exploratory phase. The factor solution was confirmed, but showed quite highly correlated latent factors. We fitted the model and tested whether or not the bifactor structure was better than the previous one (four correlated factors). Actually, we had evidence supporting the presence of a general factor, providing a measure of disgust susceptibility, along with the four specific factors previously defined. This result could be useful also from the clinical perspective since the DPSS-R questionnaire will be used in clinical context, where underlying factors may be related to different and specific psychopathological profiles. Finally, we examined and visualized the interrelationships among the four DPSS-R factors and the external scales (Anxiety Sensitivity, Disgust Scale and Padua) using a graphical model approach.

## Introduction

Although emotional regulation represents a main topic in psychiatric research, the emotion of disgust received less attention and, until the 90s, disgust processing has been under-investigated (Phillips et al., 1998). In the last decade, only few descriptive and experimental studies have examined the role of disgust in the development and maintenance of several psychopathological conditions such as Hypochondriasis (Davey and Bond, 2006), Social Phobia (Montagne et al., 2006), Specific Phobias (Olatunji, 2006; Olatunji et al., 2006), contamination-based Obsessive-Compulsive Disorder (Olatunji, 2010), Anorexia Nervosa (Aharoni and Hertz, 2012) and Psychotic Disorders (Schienle et al., 2003).

The Disgust Scale (Haidt et al., 1994) and the Disgust Questionnaire (Rozin et al., 1984) were developed within psychometric studies, assessing disgust propensity (DP) in very specific contexts with a lack of generalization of the construct itself (Tolin et al., 2006). In order to overcome this limitation, some authors proposed the Disgust Propensity and Sensitivity Scale (DPSS), which measures the DP and disgust sensitivity irrespective of disgust elicitors (Cavanagh and Davey, 2000). The revised version (DPSS-R, van Overveld et al., 2006) consists of 16 items (rated on a 5-point Likert scale) divided into two subscales (each consisting of eight items) which assess respectively DP and Disgust Sensitivity (DS). In the context of psychopathology, DP is described as a *general tendency to respond with the emotion of disgust to any given situation* (van Overveld et al., 2006), while DS refers to the unpleasant feeling of experiencing the emotion of disgust.

van Overveld et al. (2006) analyzed the statistical properties of DPSS-R on a large sample of Dutch students showing that the questionnaire has an adequate internal consistency: confirmatory factor analysis (CFA) of the DPSS-R supported a two-factor model consisting of DP (*α* = 0.78; test–retest reliability = 0.69) and DS (*α* = 0.77; test–retest reliability = 0.77). Olatunji et al. (2007) administered the test on a large non-clinical sample of undergraduate American students showing that DPSS-R has a good reliability and convergent validity. The presence of two underlying factors, i.e., DP and DS, was found using a principal components analysis. Although the studies conducted by van Overveld et al. (2006) and Olatunji et al. (2007) are similar in terms of the number of underlying factors, there are two items which loaded on the DP factor in the study by van Overveld et al. (2006) that loaded on the DS factor in the factor solution proposed by Olatunji et al. (2007) and other two items for which the opposite applies. It was proposed that these conflicting findings were related to cross-cultural differences. Fergus and Valentiner (2009), in a study carried out in two large independent samples of non-clinical United States College students, confirmed that the DPSS-R contains two distinguishable factors, measuring DP and DS. However, they proposed a reduced version of the DPSS-R (namely, DPSS-12), suggesting the removal of four problematic items (items 4, 9, 12, and 13) in order to obtain more reliable scales. Goetz et al. (2013) examined the factor structure of the DPSS-12 in two large, non-clinical United States student samples. Exploratory and confirmatory factor analyses were carried out revealing the presence of three factors. The Authors named the third factor “self-focused/ruminative disgust,” which consistently correlated with measures of obsessional symptoms. Moreover, they interpreted this factor observing that subjects, showing self-focused and ruminative behaviors when feeling disgusted, may also have difficulties in controlling unwanted thoughts.

More recently, Iwasa et al. (2016), while examining the psychometric properties of the Japanese version of the DPSS-R questionnaire, found an identical solution to the English version proposed by Olatunji et al. (2007), using both an exploratory and a confirmatory approach.

To the best of our knowledge, Dassisti et al. translated the DPSS-R in Italian and the translation was approved by Dr. van Overveld^1^. However, the questionnaire has not been validated yet. Soto et al. (2016) recently suggested that cultural differences could lead to divergent emotional regulation. The Authors investigated disgust (down and up) regulation during the vision of disgusting videos with interesting but preliminary findings and they concluded that future work should deepen their analysis including specific measures about emotion values and beliefs. Following this suggestion and due to the reported controversial findings, which we found in literature, we aimed at validating the Italian version of the DPSS-R questionnaire, and investigated the factor structure and the psychometric properties of the instrument.

## Study 1: Exploratory Analysis

### Sample Description and Procedure

Two hundred eighty five out of 328 questionnaires were completely filled out and were considered in the analyses (56.5% females and 43.5% males, average age 30.02 years, ranging from 18 to 66 years, average years of schooling 16.88).

DPSS-R questionnaire consists of 16 items aiming at assessing two distinct constructs, i.e., DP and DS. Each statement is rated on a 5-point Likert scale (from 1 “never” to 5 “always”). The complete item description in Italian and English, retrieved from http://www.markvanoverveld.com/research-indices/, is provided in **Table 1**. Dr. van Overveld provided us with this version and granted the permission to reproduce the items of DPSS-R, confirming that the questionnaire on his website was without any copyright restrictions. Dassisti, Fagliarone, Questa, Catarinella, Oliviero, Pugliese, and Cosentino translated the DPSS-R in Italian. The questionnaire was administered through an online questionnaire, developed with the help of System Administrators of Vita-Salute S. Raffaele University, and a web link was then provided to allow online access. The link was promoted by word of mouth, on social networks, University website and project website^2^. This online survey strategy was implemented in order to obtain data from a larger sample. Instructions for filling in the questionnaire were provided, along with a brief description of the project and information on planned retest procedure.

**Table 1:** Disgust Propensity and Sensitivity Scale-Revised (DPSS-R) questionnaire with complete item description in Italian and English retrieved from http://www.markvanoverveld.com/research-indices/.

Item ID	Description (Italian/English)
D1	Evito le cose disgustose
	I avoid disgusting things
D2	Quando provo disgusto temo di svenire
	When I feel disgusted, I worry that I might pass out
D3	Provare nausea mi spaventa
	It scares me when I feel nauseous
D4	Penso che le cose disgustose possano causarmi una malattia/infezione
	I think disgusting items could cause me illness/infection
D5	Mi sento disgustato
	I feel repulsed
D6	Le cose disgustose mi danno il volta stomaco
	Disgusting things make my stomach turn
D7	Faccio una smorfia di disgusto
	I screw up my face in disgust
D8	Quando provo nausea ho paura di vomitare
	When I notice that I feel nauseous, I worry about vomiting
D9	Quando provo disgusto è una sensazione intensa
	When I experience disgust, it is an intense feeling
D10	Provo disgusto
	I experience disgust
D11	Quando mi sento svenire mi spavento
	It scares me when I feel faint
D12	Provo disgusto più facilmente rispetto agli altri
	I become disgusted more easily than other people
D13	Mi preoccupa ingerire qualcosa di disgustoso
	I worry that I might swallow a disgusting thing
D14	Trovo alcune cose disgustose
	I find something disgusting
D15	Mi imbarazza provare disgusto
	It embarrasses me when I feel disgusted
D16	Penso che provare disgusto mi faccia male
	I think feeling disgust is bad for me


*Dassisti, Fagliarone, Questa, Catarinella, Oliviero, Pugliese, and Cosentino translated the DPSS-R in Italian. Item description found in van Overveld et al. (2006) and Olatunji et al. (2007) was used to reproduce the Table. Participant were asked to read and rate each statements indicating how often (from never to always) it applies to them*.

### Psychometric Measurements

The Disgust Propensity and Sensitivity Scale-Revised (DPSS-R, van Overveld et al., 2006) comprises 16 items out of the 32 initially proposed by Cavanagh and Davey (2000) and included in the DPSS. The selection of these 16 items was led by “theory- and data-drive considerations” (van Overveld et al., 2006, p. 1246). Items were rated on a 5-point Likert scale (from 1 “never” to 5 “always”) and participants were asked to select the response option which best applied to them. The questionnaire was originally developed in Dutch.

Since the original questionnaire was developed to measure DP and DS, van Overveld et al. (2006) performed an exploratory factor analytic study (principal component analysis with Oblimin rotation), fixing a two-factor solution. The solution accounted for 35.6% of total variance and the two factors DP and DS were moderately correlated (*r* = 0.54). The CFA of the DPSS-R, carried out on the polychoric correlation matrix of the data, supported the two-factor model. Latent factors resulted highly correlated (*r* = 0.78). With respect to the reliability, van Overveld et al. (2006) found that Cronbach’s α were equal to 0.78 for the DP scale and to 0.77 for the DS scale. Moreover, test–retest reliability measured through Intraclass correlation coefficient (ICC) was equal to 0.77.

Although with a different item-factor structure, a two-factor solution emerged also in the exploratory factor analysis (i.e., a principal component analysis with Oblimin rotation) carried out by Olatunji et al. (2007) when using the English version of the questionnaire. The evaluation of the scree plot and the factor interpretability drove the choice of this solution. The solution accounted for 49.3% of total variance, with the first factor explaining a substantial portion (41.3%). In this case, the correlation among factors was 0.66.

Recently Iwasa et al. (2016), examined the psychometric properties of the Japanese version of the questionnaire and found an identical solution to the English version proposed by Olatunji et al. (2007), using both an exploratory (maximum likelihood extraction method and Oblimin rotation) and a confirmatory approach (maximum likelihood estimation was applied). The number of factors to retain in the exploratory factor analysis was determined based both on the scree plot examination and parallel analysis. The solution accounted for 46.15% of the total variance. DP and DS factors were positively correlated (*r* = 0.67). Cronbach’s α of the DP and DS subscales were 0.86 and 0.79, respectively. The test–retest reliability was adequate (ICC coefficient was equal to 0.72 for DP and to 0.75 for DS).

### Statistical Methods

Exploratory factor analysis for ordinal Likert-type items was carried out on the matrix of polychoric correlations to examine the dimensional structure underlying the DPSS questionnaire. In the presence of ordinal data, factor analysis should be carried out on the matrix of polychoric correlations matrix rather than on Pearson’s thus resulting in a more accurate reproduction of the original correlation structure (Holgado-Tello et al., 2010).

In order to select the number of factors to retain in exploratory factor analysis, several indices have been considered (Courtney, 2013). In particular, we run Horn’s Parallel Analysis (Horn, 1965) and evaluated the Velicer’s Minimum Average Partial criterion (MAP; Velicer, 1976), the Very Simple Structure criterion (VSS; Revelle and Rocklin, 1979) and the empirically derived Bayesian Information Criterion (eBIC; Revelle, 2016). The total amount of variance explained, the factor loadings magnitude and the reproduced correlation matrix were also examined. The choice of the final factorial solution was based on the criteria of parsimony and interpretability.

To assess test–retest reliability for the total score, ICC was estimated using Linear Mixed Effects (LME) models for repeated measures. Roughly, ICC provides a measure of the “average” correlation between the total DPSS-R scores obtained in the two different time occasions (e.g., test administrations) within the same cluster, represented, in our case, by participant ID.

All the analyses were performed using R statistical software (R Development Core Team, 2015). In particular, for exploratory factor analysis, *psych* package (Revelle, 2016) was used, along with *nFactors* package (Raiche, 2010) for choosing the appropriate number of factors to be retained in the analysis. ICC was estimated by means of the *CorrMixed* package (van der Elst et al., 2016).

### Results

#### Identifying Underlying Factors

**FIGURE 1:**
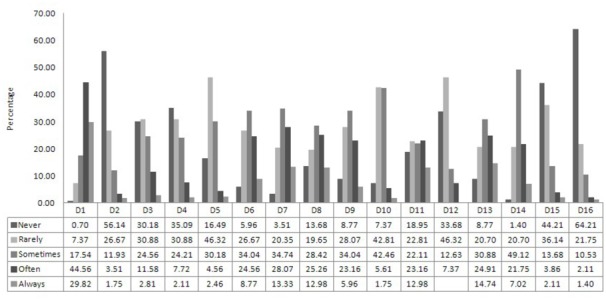
**Percentage distribution of the ratings provided for each item of the DPSS-R questionnaire.** D1–D16 indicates the ID of the questionnaire items. The complete list of DPSS-R item statements is provided in **Table 1**, both in Italian and English. Each item was rated in a five-point Likert scale (from 1 = Never to 5 = Always).

The frequency distribution of ratings provided for each DPSS-R item is shown in **Figure 1**. Sequential identification labels (D1-D16) were used to denote DPSS-R items.

Parallel analysis suggested a five-factor solution. Velicer’s MAP criterion indicated a one-factor solution as best and empirical Bayesian Information Criterion (eBIC) suggested 4 factors. VSS criteria (complexity 1, complexity 2) suggested the extraction of fewer factors, namely 1 or 2 factors. However, it has been reported in the literature that VSS criterion is not always optimal and simulation results have shown that this criterion is not appropriate especially when data shows a complex factor structure (Revelle, 2016). Based on these criteria and considering that a three factor solution has been proposed in literature by Goetz et al. (2013), we explored a range of solutions going from 2 to 5 factors.

Finally, considering the total amount of variance explained, the factor loadings magnitude, ability of the solution to reproduce the original correlation, privileging aspects related to parsimony and interpretability, we chose a four-factor solution as best.

For the sake of interpretability, an oblique rotation was used thus allowing factors to correlate with each other. Ordinary Least Squares procedure aiming at minimizing the residual (off-diagonal) correlation matrix (*minres* algorithm) was applied to extract factors. The Root Mean Square of the Residuals for the selected factor model was 0.04. Four factors explained 46% of the variance. Factors showed inter-correlations in the range of 0.24–0.36, thus supporting the choice of an oblique rotation.

**FIGURE 2:**
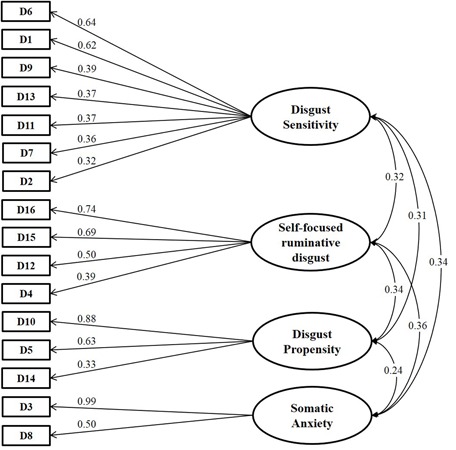
**Diagram showing standardized loadings (pattern matrix) and correlations among factors obtained in the exploratory factor analysis for ordinal data.** DPSS-R items are denoted by sequential labels, from D1 to D16. Item statements are listed in **Table 1**, both in Italian and English.

The factor containing the four items D4, D12, D15, and D16 was related to the *self-focused/ruminative disgust* (SFR). The factor including the items D5, D10, and D14 was labeled DP. The factor containing the items D3 and D8 was labeled *somatic anxiety* (SA). The last factor including seven items (D1, D2, D6, D7, D9, D11, D13) was named DS. A graphical representation of the emerging structure is shown in **Figure 2**. Item statements are listed in **Table 1**.

#### Test–Retest

Subjects taking part into the first phase of the study were free to submit their email address to be contacted for the retest phase. Out of the 285 subjects that completed the questionnaire, 112 participants (62.5% females and 37.5% males, average age 32 years, ranging from 18 to 66 years, average years of schooling 16.98) gave their permission to be contacted and completed the questionnaire 1 month later.

Intraclass correlation coefficient for the total score was equal to 0.73 (95% CI: [0.64, 0.81]). Following indications provided in the literature (e.g., Nunnally and Bernstein, 1994; Terwee et al., 2007; Paiva et al., 2014), test–retest values larger than 0.70 are considered adequate.

## Study 2: Confirmatory Analysis And Convergent Validity

### Sample Description

CFA for ordinal data was then applied in an independent non-clinical sample to determine whether the identified factorial structure holds. Two hundred ninety three participants (56% females and 44% males, average age 29.55 years, ranging from 18 to 81 years) were enrolled as a test set to validate the factor structure obtained in the exploratory phase and completed the DPSS-R questionnaire. Participants completed three additional questionnaires examining constructs related to DP and sensitivity and including the Anxiety Sensitivity Index (ASI), the Disgust Scale and the Padua Inventory (PI).

### Psychometric Measurements

The PI is a self-report questionnaire, which consists of 60 items. It describes the most common obsessional and compulsive behaviors related to Obsessive–Compulsive Disorder. Subjects are required to mark their agreement on a 5-point Likert scale, which ranges from 0 (i.e., “Strongly Disagree”) to 4 (i.e., “Strongly Agree”). Results are divided into 5 scores: a global score and 4 factors scores (i.e., Factor 1-“Impaired control of mental activities”; Factor 2-“Becoming contaminated”; Factor 3-“Checking behaviors”; Factor 4-“Urges and worries of losing control over motor behaviors”). As a non-diagnostic questionnaire, there is no cutoff for any of the factors, but higher scores mean more severe symptoms. The Cronbach’s α for the whole questionnaire was 0.94 and it varied from 0.70 to 0.90 for each subscale (Sanavio, 1988).

The ASI (Reiss et al., 1986) is a 16-item self-report questionnaire designed to assess the dispositional tendency to fear the somatic and cognitive symptoms of anxiety due to a belief that these symptoms may be dangerous or harmful. Each item is rated on a 5-point Likert scale ranging from 0 (very little) to 4 (very much).

The Disgust Scale (Haidt et al., 1994) is a 32-item self-report scale that was developed to measure individual differences in sensitivity to disgust. It consists of eight subscales: seven domains of disgust elicitors and one on magical beliefs, related to magical thinking and entailing beliefs of implausible contamination. Each domain of the DS is examined through four questions: the first two items are true/false questions, while the other remaining two items are answered on a 3-point Likert scale (from 0, “not disgusting at all” to 1, “extremely disgusting”). A total score, ranging from 0 to 32 (higher values are indicative of greater DS), may be computed along with subscale scores. Cronbach’s αs for the eight subscales are poor and range from 0.34 to 0.64 (Haidt et al., 1994), while α for the total score is high (0.84; Haidt et al., 1994).

### Statistical Methods

Confirmatory factor analysis was performed using *cfa* function in the *lavaan* package (Rosseel, 2012). For CFA, Diagonally Weighted Least Squares (DWLS) estimator was used along with a robust method to compute the standard errors. Actually this estimation method has become very popular for carrying out ordinal factor analyses, since it allows solving efficiently non-convergence problems (Forero et al., 2009).

To evaluate the goodness of fit of the model, the chi-square test statistic was calculated. Non-significant values indicate that the model fits the data well. Chi-square estimations are strongly affected by the sample size. Models fitted on large data sets often result in significant chi-square tests due to the sample size, which may lead to possible mistakes in concluding about model fit (Ullman, 2001; Schumacker and Lomax, 2004; Jackson et al., 2009; Byrne, 2010). Moreover, the normed chi-square, i.e., the ratio between the chi-square statistic and its degrees of freedom, was evaluated. Although there is no consensus on the threshold value indicating goodness of fit, values ranging from less than 2 (Ullman, 2001) to less than 5 (Wheaton et al., 1977; West et al., 2012) are considered acceptable.

As suggested in Hu and Bentler (1999), several other indices have been considered to select the final model. In particular, in addition to the above mentioned chi-square statistic, we examined and reported the Goodness-of-Fit Index (GFI), the Adjusted Goodness-of-Fit Index (AGFI), the Root Mean Square Error of Approximation (RMSEA), the Weighted Root Mean Square Residual (WRMR), the Comparative Fit Index (CFI), the Non-Normed Fit Index (NNFI, also known as the Tucker-Lewis Index). GFI ranges from 0 to 1 and values greater than 0.90/0.95 indicate good fit (Miles and Shevlin, 1998; Hooper et al., 2008). AGFI adjusts GFI accounting for model complexity (i.e., it is adjusted for the degrees of freedom) and has the same theoretical range (from 0 to 1, with larger values indicating better fit). For RMSEA values of 0 indicate perfect fit and values close to 0.06 or below are indicative of good fit (Hu and Bentler, 1999). WRMR values less than 1 can be interpreted as indicator of good model (Yu and Muthén, 2001). CFI ranges from 0 to 1, with values closer to 1 implying good model fit. Since it is non-normed, NNFI may take values outside the 0–1 range. CFI and NNFI values in the range of 0.90–0.95 indicate that the model fit is acceptable (e.g., Bentler, 1990). For the sake of interpretation and following recommendation in the literature (West et al., 2012), RMSEA value was supplemented with a confidence interval (CI). In presence of good fit, the suggested lower limit of the RMSEA’s CI falls at or below 0.05 and upper limit should not exceed 0.10. Morever, GFI^∗^ (a.k.a. gamma hat, Steiger et al., 1985; Maiti and Mukherjee, 1990) and AGFI^∗^ (a.k.a., adjusted gamma hat, Steiger et al., 1985; Maiti and Mukherjee, 1990) indices were evaluated. Differently from GFI and AGFI, the modified versions of these indices, i.e., GFI^∗^ and AGFI^∗^, are not sensitive to the sample size. Moreover, AGFI^∗^ has the key advantage over GFI^∗^ of including penalty for model complexity (West et al., 2012). Both GFI^∗^ and AGFI^∗^ ranges from 0 to 1 (actually also negative values can be obtained as an effect of serious model misspecification) with larger values indicating better fit: while for GFI^∗^ a cutoff of 0.95 has been proposed in the literature (Hu and Bentler, 1998), no cutoff criteria have been proposed for AGFI^∗^ (West et al., 2012).

Bifactor models represent an alternative modeling strategy, particularly useful when high correlations among factors emerge in the confirmatory phase, allowing to represent general constructs comprised of several highly related domains (Chen et al., 2006). The main feature of this model is that it allows simultaneously evaluating both the degree with which items reflect a single common factor and in which they measure specific domains. The presence of a general factor was evaluated through a test for comparing suitable nested model. Factor loadings were interpreted based on Thurstone’s recommendation, i.e., values equal to or larger than 0.30 are considered salient. The degree of essential unidimensionality was examined by means of Explained Common Variance (ECV) (Ten Berge and Sočan, 2004; Reise et al., 2010; Rodriguez et al., 2016). ECV for the general factor is simply calculated, using the estimated factor loadings, as the ratio of variance explained by the general factor divided by the variance explained by the general plus the group factors (Reise, 2012), where group factors are assumed to be uncorrelated. High ECV values are indicative of the unidimensionality of the construct, hence suggest the presence of a general factor prevailing on group factors model.

Ordinal *α* coefficient was calculated to assess internal consistency (Zumbo et al., 2007). This version of the coefficient *α* is preferred in presence of ordinal data, providing more accurate evaluation. Feldt’s (1965) method was used to compute the CI.

A graphical model (Hojsgaard et al., 2012) was finally used to analyze and provide a graphical representation of the relationships between the DPSS-R scales and the external measurements of ASI, Disgust Scale and PI. Actually, this type of modeling is becoming more and more popular also in biomedical and clinical research since it facilitates the description and visualization of the underlying patterns in complex multivariate data sets. Going beyond traditional bivariate approaches (e.g., correlation coefficients), graphical models are multivariate statistical models that enable to explore the dependence structure among variables, under some conditional independence constraints encoded in a graph (Lauritzen, 1996). Hence, they could be used in a preliminary exploratory phase also as a valid and effective alternative to traditional Structural Equation Modeling (SEM) approaches, which instead require the prior specification of the dependency structure (Trentini et al., 2015). In graphical models, each node in the graph represents a variable and edges represent conditional dependency between variables. Missing edges between nodes mean that the variables are conditionally independent given the remaining variables. Here, the model was fitted using the *gRapHD* package in R (Abreu et al., 2010) designed to obtain acyclic undirected graphs. The package uses the algorithm proposed by Chow and Liu (1968) to find a graph containing no cycles that minimizes a criterion and here the selected criterion was the BIC.

All statistical analyses have been performed in R statistical software (version 3.2.3).

### Results

#### Comparing Alternative Models

Four alternative models were tested using CFA carried out on the polychoric correlation matrix. We allowed underlying factors to be correlated. In addition to the simplest unidimensional model, we estimated the two-factor model originally proposed by van Overveld et al. (2006), the two-factor model identified by Olatunji et al. (2007) and the four-factor model obtained in the exploratory analysis.

All chi-square tests were significant, suggesting that none of the models fits the data. However, we are aware that chi-square estimations are strongly affected by large sample size. The normed chi-square was closer to the threshold of 2 (Ullman, 2001) for the four-factor solution. However, all the tested models have a normed chi-square lower than the other threshold of 5, proposed by Wheaton et al., 1977 and West et al. (2012).

**FIGURE 3:**
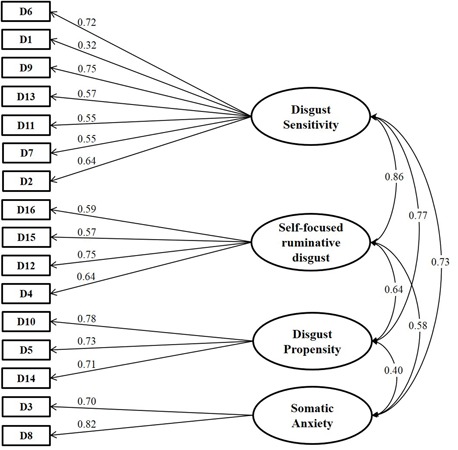
**Standardized path coefficients for the four-factor model estimated in the confirmatory factor analysis.** Values in the curved double-headed arrows indicates the correlations among factors. Standardized parameter estimates of the CFA model are shown in the directional arrows linking the underlying factors to the DPSS-R questionnaire items. DPSS-R items are denoted by sequential labels, from D1 to D16. Item statements are listed in **Table 1**, both in Italian and English.

In general, considering the other fit indices, the four-factor solution resulted slightly better than the other: in particular, RMSEA was lower and equal to the cutoff value 0.06, GFI and AGFI values were higher than those obtained in the single-factor and two-factor solutions, equal respectively to 0.98 and 0.97. The same consideration holds for CFI and NNFI that were closer to 1 in the four-factor solution than in the other tested models. In the four-factor solution, WRMR was smaller and equal to 1.05 (slightly higher than the recommended threshold of 1.0). All the results are reported in **Table 2**. The estimated model is shown in **Figure 3**.

**Table 2:** Goodness of fit indices for the compared factor models.

Model	χ^2^	df	χ^2^/df	*p*-value	CFI	NNFI	WRMR	RMSEA (90% CI)	GFI	AGFI	GFI^∗^	AGFI^∗^
One factor	346.57	104	3.33	<0.0001	0.95	0.94	1.37	0.09 (0.08–0.1)	0.97	0.95	0.91	0.88
Two factors (a)	293.62	103	2.85	<0.0001	0.96	0.96	1.26	0.08 (0.07–0.09)	0.98	0.96	0.92	0.9
Two factors (b)	298.78	103	2.9	<0.0001	0.96	0.95	1.27	0.08 (0.07–0.09)	0.97	0.96	0.92	0.9
Four factors	203.44	98	2.08	<0.0001	0.98	0.97	1.05	0.06 (0.05–0.07)	0.98	0.97	0.96	0.94
Bifactor model	151.1	88	1.72	<0.0001	0.95	0.93	1.05	0.05 (0.04–0.06)	0.94	0.91	0.97	0.96


*In the Table, (a) refers to the two factors solution proposed by van Overveld et al. (2006) and (b) to the two factors solution proposed by Olatunji et al. (2007). Indices for the bifactor models are also reported*.

Unlike the exploratory phase, the latent factors are quite highly correlated with each other. Similar results have been reported in the literature (van Overveld et al., 2006; Olatunji et al., 2007; Fergus and Valentiner, 2009). Actually, the high correlation among latent factors may indicate the presence of a general factor running through all the items (Reise et al., 2007; Reise, 2012). Hence, accordingly, we estimated a bifactor model, which allows to represent a general construct comprised of several highly related domains (Chen et al., 2006). By using standard constrains on the parameters for model identifiability, we fitted the model and its fit statistics are reported in **Table 2**. We may observe that the bifactor model is the best model for many of the indices. We also tested whether or not we may hypothesize the presence of a general factor along with the four specific domains and we had evidence supporting the presence of a general factor (*p* < 0.0001).

All the items on the general factor had salient loadings. Nine out of the 16 items have a loading on the general factor of at least 0.50 in absolute value. These items are mostly from DS and DP content domains (D2, D4, D6, D7, D8, D9, D12, D13, and D14). Regarding the group factors, salient loadings were found for one out of seven DS items (-0.81 for D2, while the others ranged from -0.02 to 0.22), for two out of four items in the SFR factor (0.77 for D15, 0.50 for D16, while the others were 0.05–0.12), for two out of three items of the DP factor (0.88 for D10, 0.39 for D5 and 0.18 for D14) and for all the SA items (0.56 for D3 and 0.52 for D8). Moreover, we wish to emphasize that items D2, D3, D8, D10, D15, and D16 had a higher loading on the group factors than on the general factor. This means that these items are more representative of the group factor than of the general factor.

As already mentioned, when fitting a bifactor model, it is important to assess the relative strength of the general factor and we assessed it with the ECV. We found that the general factor accounted for 57.85% of the common variance. Group factors for DS, SFR, DP and SA accounted for 9.96, 11.58, 12.77, and 7.84% of the common variance, respectively. Thus, together, the group factors accounted for 42.15% of the common variance. In the bifactor model all the items significantly load on the general factor. With the exception of items D6, D9, D11, D12, and D4, all the items loaded significantly on their specific factors.

Hence, we may conclude that there could be one general factor providing a measure of disgust susceptibility along with four domain specific factors (reflecting self-focused disgust, DP, somatic anxiety and DS). Further studies are needed to investigate whether both a multidimensional and an unidimensional representations may co-exist for the DPSS-R questionnaire.

#### Convergent Validity

**FIGURE 4:**
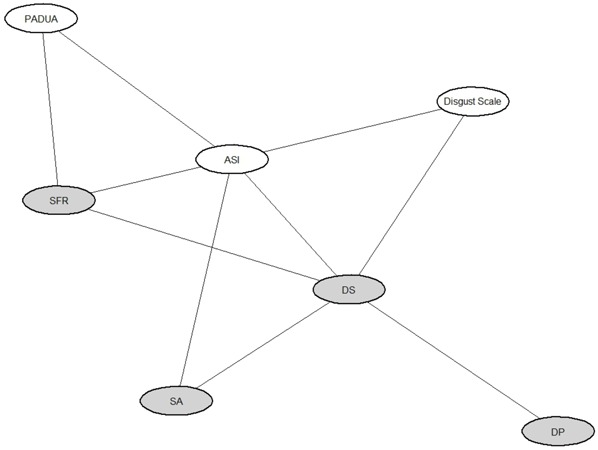
**Graphical model showing the association structure among DPSS-R scales and external psychometric measurements.** Gray ellipses represent the four scales identified and confirmed by factor analyses. In particular, SFR is the self-focused/ruminative disgust, DP is the disgust propensity, SA indicates the somatic anxiety and DS the disgust sensitivity. In white ellipses are represented the Anxiety Sensitivity Index (ASI), the Disgust Scale and the Padua Inventory (PI) used as external scales.

To examine and visualize connections between the four scales (SFR, DS, DP, SA) and the external scales (ASI, Disgust Scale and PI) we fitted a graphical model. In particular, an acyclic undirected graph was derived. This tool allowed us to highlight (i) a relationship between SFR and the obsessive traits measured by PI and (ii) the associations between DS, the Disgust scale and DP. As mentioned above, DP and DS are associated even in our solution. Moreover, SA is connected to ASI and DS. Results are shown in **Figure 4**.

#### Reliability

The internal reliability coefficient, measured by the ordinal *α* coefficient, for the DPSS-R questionnaire was 0.88 (95% CI: [0.85, 0.9]). Thus, the DPSS-R total score demonstrated good internal consistency. The corrected item-to-total correlation for the items ranged from 0.27 to 0.71.

Reliability for each scale was also examined. In particular, for the scale containing items D4, D12, D15, D16 and to the *self-focused disgust*, the ordinal *α* was equal to 0.73 (95% CI: [0.67, 0.78]), with no changes in this coefficient after the removal of each single item. The corrected item-to-total correlation for the items in this scale ranged from 0.50 to 0.72. For the scale containing items D5, D10 and D14 (labeled *DP*), the ordinal *α* was equal to 0.70 (95% CI: [0.63, 0.75]), with item D14 increasing *α* to 0.75 after removal. The corrected item-to-total correlation for the items in this scale ranged from 0.46 to 0.76. The ordinal *α* of the scale including item D3, D8 (related to *anxiety traits* in the experience of disgust) was equal to 0.71 (95% CI: [0.64, 0.77]). The corrected item-to-total correlation for the items in this scale was equal to 0.65. Finally, the ordinal *α* of the last scale including items D1, D2, D6, D7, D9, D11, and D13 (named *DS*) was equal to 0.77 (95% CI: [0.72, 0.81]). The corrected item-to-total correlation for the items in this scale ranged from 0.36 to 0.73. Hence, we may conclude that for the scales there was an acceptable internal reliability.

## Discussion

Disgust is a complex emotion playing an important role in the development and maintenance of several psychopathological conditions. Nowadays, the development of adequate assessment tools providing a dimensional evaluation of disgust’s features (regardless of context) is pivotal to enhance research in this area. van Overveld et al. (2006) exhorted researchers to better investigate the emotion of disgust, its pathological processing and how it affects human behavior. Identifying other decontextualized indices could help to improve the understanding and characterization this emotion.

In this paper, we examined the psychometric properties of the Italian version of DPSS-R in two non-clinical Italian samples and we validated the questionnaire. Four latent constructs emerged from our analysis: (a) self focused disgust, (b) DP, (c) somatic anxiety and (d) DS. The proposed factor solution provided an acceptable model fit across multiple indices when compared to the other alternative models using both an exploratory and a confirmatory analysis.

It’s crucial to underline that we found quite high correlations among latent factors in the confirmatory analysis: this result is in line with previous studies. van Overveld et al. (2006) found that the latent factor correlation was high (*r* = 0.78), Olatunji et al. (2007) in the exploratory analysis found that DS and DP were also highly correlated with each other (*r* = 0.66). Fergus and Valentiner (2009), in the confirmatory analysis carried out in the reduced-item version of the questionnaire, identified the two factors and reported that a correlation of 0.72 between the two latent factors.

The bifactor model analysis allowed us to show that in our general population a main factor, that we called “Disgust Susceptibility,” could explain along with the four factors, how subjects deal with disgust processing. The interpretation of the four factors may be of interest especially when administering the psychometric tool in a clinical population. More in detail, the fact that the proposed constructs were highly associated is not surprising because they would represent different, but integrated, responses to disgust. In this perspective “Disgust Susceptibility” could be sufficient to describe a healthy subject that tends to respond to emotion of disgust to any situation, feeling more disgusted, with high somatic anxiety and with more ruminating thoughts. Furthermore, disgust has been linked with many anxiety disorders, which are commonly characterized by two main symptom clusters: excessive and uncontrollable worries and somatic symptoms (American Psychiatric Association, 2013). We expect that the proposed factor structure may allow researchers to examine common features and dissimilarities along anxiety spectrum disorders with a special focus on cognitive and somatic characteristics of disgust. Under this perspective, the proposed factors represent four specific dimensions to process disgust; in the general population, they are highly interrelated. Therefore, including the evaluation of psychopathological conditions may allow deriving more specific psychopathological profiles. Our proposal is reinforced by the association structure emerging from the graphical model, which we discuss below within each factor description.

The factor including items D4, D12, D15, and D16 was called “self-focused/ruminative disgust” consistently with Goetz et al. (2013), which found similar results on the shorter version of the revised questionnaire (DPSS-12). This factor includes four items reflecting negative thoughts toward the experience of feeling disgusted. *Rumination* is a repetitive, iterative and somewhat uncontrollable thought process. Moreover, it is typically ego-dystonic and focused on past events and experiences (Davey et al., 2003). Our results suggest that self-focused/ruminative disgust is a distinct component of disgust processing strongly linked with ruminative thoughts. This result is reinforced by the graphical model analysis, which shows a distinctive pattern of association with obsessive-compulsive symptoms’ dimension. Since the unique real distinction between obsession and rumination is that obsessions lead to compulsions (Davey et al., 2003), future studies are recommended to clarify whether self-focused/ruminative disgust is a specific disgust feature that characterizes cleaning/washing OCD patients (Olatunji, 2010). A possible approach is to investigate and compare self-focused/ruminative disgust’s behavioral outcome in washers compared with other OCD subtypes and patients with other anxiety symptoms.

The factor consisting of items D5, D10, and D14 and named “DP” indicates a *general tendency to respond with the emotion of disgust to any given situation* (van Overveld et al., 2006). The three items loading on this factor are related with the subject’s self-reported frequency to “feeling disgusted” or to “taste disgust.” These three items were already included as measures of DP in the original version of the questionnaire. Not included items (if compared with original version) seem to refer to other factors and constructs. In fact, the items “I avoid disgusting things/Evito le cose disgustose,” “Disgusting things make my stomach turn/Le cose disgustose mi danno il volta stomaco,” “When I experience disgust, it is an intense feeling/Quando provo disgusto è una sensazione intensa,” “I screw up my face in disgust/Faccio una smorfia di disgusto” seem to be more related to the concept of DS, which refers to subject’s discomfort caused by the experience of disgust. Indeed, avoidance behavior, involuntary facial expression and experiencing intense feelings are strictly related with the subjective bad feeling of being disgusted. The analysis of frequency distribution of the ratings provided for the latter three items (item D6, D7, and D9), in our sample, revealed a similar trend in the responses, with about 34% of the participants answering “sometimes.” Moreover, almost no one (0.7% of the participants) answered “never” to the first item, e.g., “Evito le cose disgustose” (“I avoid disgusting things”). In addition, for this item, we observed the highest percentage of responses “always,” with respect to all the items. The item “I become disgusted more easily than other people/Provo disgusto più facilmente rispetto agli altri” is more associated to a thought process than to the probability to experience disgust. In our survey, nobody answered “always” to this item. We recall that this item has been highlighted as “problematic” and, as a consequence, removed in the revised version of the questionnaire proposed by Olatunji et al. (2007) since it was characterized by questionable face validity and cross-cultural factor instability (actually, it loaded on the DS factor rather than on the DP factor as in van Overveld et al., 2006).

The factor labeled “somatic anxiety” consisting of items D3 and D8 was associated with anxiety sensitivity. This construct refers to the misattribution of bodily sensations related to anxiety as a harmful experience causing more intense anxiety or fear. This is also associated with DS and this result is in line with findings emerging from literature. In fact, Woody and Tolin (2002) suggested that trait anxiety and disgust are strongly related because they are both partially mediated by neuroticism. Instead, other authors suggested considering them as independent constructs, thus emphasizing the presence of a main factor referring to psychopathological anxiety (Davey and Bond, 2006). In this perspective, the factorial structure emerging from our data highlights this functional separation between constructs, showing their interrelationship. Somatic anxiety may reflect somatic vulnerability toward the experience of anxiety, which could be related with the experience of feeling disgust.

The factor including all the remaining items is “DS,” which refers to the unpleasant feeling of experiencing the emotion of disgust. Items loadings on this factor predominantly reflect worried/avoidant behavior and autonomic arousal over feeling disgust.

By revealing a self-focused/ruminative component of disgust and emphasizing the role of somatic anxiety traits in the experience of disgust, the proposed factor model could improve the evaluation of the individual’s tendency to process disgust information in a way that is generalizable across diverse contexts and across different clinical population.

The identification of new constructs may allow overcoming some difficulties experienced when studying a complex and multifaceted emotion in psychopathological field (Berle and Phillips, 2006). Authors suggested to integrate physiological aspects (in particular, patterns of sympathetic nervous system activity) with cognitive and appraisal aspects in the evaluation of disgust. In line with these recommendations, the proposed factor solution allows examining somatic, cognitive and appraisal aspects of disgust with a short and easy to administer self-report. Moreover, we are planning experimental sessions to monitor the physiological response induced by disgust eliciting stimuli. By integrating our findings with behavioral and physiological measurements, we aim at providing a better understanding of basic dimensions of functioning underlying normal and abnormal disgust processing as stated by recent advanced in psychopathological research (Insel et al., 2010).

Hence, future researches should focus also on the identification and characterization of specific disgust profiles in clinical populations trying to answer questions, such as, “Is it true for each clinical population that disgust responses occur at both a primary (DP) and a secondary (DS) level (van Overveld et al., 2006)?” SFR and SA, in our opinion could help to answer this question.

As already mentioned in the Section “Introduction” and throughout the paper, it has been suggested that controversial findings in characterizing disgust experience may be attributed to cross-cultural differences (Olatunji et al., 2007; Soto et al., 2016). Also the factorial solution proposed in this paper may reflect a culture-specific way of processing, recognizing, conceptualizing, and verbalizing the disgust. Hence, up to now, its validity is limited to an Italian general population. However, our proposal should benefit from future studies carried out in clinical populations or even in different groups of healthy subjects to assess and establish measurement invariance of the questionnaire. Furthermore, given the existence of contrasting cultural norms and different ways of disgust processing and expression, it is crucial for future studies to assess the effects of disgust regulation by examining differences in the elicited physiological response and its relationship with cultural factors. These findings could help clarify cultural specific mechanisms in the expression of the emotion of disgust. Moreover, once identified ethnic and cultural specific aspects, results may be used to shape the treatment of those psychiatric syndromes that are supposed to be related with an aberrant disgust processing (i.e., OCD).

## Ethics Statement

All authors declare that all the procedures performed in this study involving human subjects were conducted in accordance with the ethical standards of the institutional and/or national research committee and with the 1964 Helsinki declaration and its later amendments or comparable ethical standards. The entire FIRB project, of which the study presented in the paper is a part, was approved by the Ethics Committee of San Raffaele Hospital. The study was carried out in a general population setting. Vulnerable populations were not involved. Participation was strictly on voluntary basis and participants were allowed to withdraw his/her participation at any time. Participants were informed that collected information would be used exclusively for this research, that was developed within the research activities of FIRB project, with the purpose of validating the Italian version of the DPSS-R. Moreover, we carried out an anonymous questionnaire survey and we didn’t collect any sensitive data compromising identities of the respondents.

## Author Contributions

RM designed data collection, contributed to interpret results, reviewing current literature on disgust and on psychometric tools to assess disgust. CB and PR designed and monitored data collection, analyzed the data and drafted the paper. CDS supervised statistical analyses and critically revised the draft paper. All authors read and approved the final manuscript.

## Conflict of Interest Statement

The authors declare that the research was conducted in the absence of any commercial or financial relationships that could be construed as a potential conflict of interest.

## Footnotes

1 http://www.markvanoverveld.com/wp-content/uploads/DPSS_ita.pdf

2 http://www.cussb.unisr.it/FIRB2012/index.html
